# The Metalloprotease, Mpr1, Engages AnnexinA2 to Promote the Transcytosis of Fungal Cells across the Blood-Brain Barrier

**DOI:** 10.3389/fcimb.2017.00296

**Published:** 2017-06-30

**Authors:** Sarisa Na Pombejra, Michelle Salemi, Brett S. Phinney, Angie Gelli

**Affiliations:** ^1^Department of Pharmacology, School of Medicine, Genome and Biomedical Sciences Facility, University of California, DavisDavis, CA, United States; ^2^Proteomics Core Facility, Genome and Biomedical Sciences Facility, University of California, DavisDavis, CA, United States

**Keywords:** AnnexinA2, Mpr1, metalloprotease, blood-brain barrier, fungal cells, *Saccharomyces cerevisiae*, mass spectrometry, *Cryptococcus neoformans*

## Abstract

Eukaryotic pathogens display multiple mechanisms for breaching the blood-brain barrier (BBB) and invading the central nervous system (CNS). Of the fungal spp., that cause disease in mammals, only some cross brain microvascular endothelial cells which constitute the BBB, and invade the brain. *Cryptococcus neoformans*, the leading cause of fungal meningoencephalitis, crosses the BBB directly by transcytosis or by co-opting monocytes. We previously determined that Mpr1, a secreted fungal metalloprotease, facilitates association of fungal cells to brain microvascular endothelial cells and we confirmed that the sole expression of Cn*MPR1* endowed *S. cerevisiae* with an ability to cross the BBB. Here, the gain of function conferred onto *S. cerevisiae* by Cn*MPR1* (i.e., Sc<Cn*MPR1*> strain) was used to identify targets of Mpr1 that might reside on the surface of the BBB. Following biotin-labeling of BBB surface proteins, Sc<Cn*MPR1*>-associated proteins were identified by LC-MS/MS. Of the 62 proteins identified several were cytoskeleton-endocytosis-associated including AnnexinA2 (AnxA2). Using an *in vitro* model of the human BBB where AnxA2 activity was blocked, we found that the lack of AnxA2 activity prevented the movement of *S. cerevisiae* across the BBB (i.e., transcytosis of Sc<Cn*MPR1*> strain) but unexpectedly, TEM analysis revealed that AnxA2 was not required for the association or the internalization of Sc<Cn*MPR1*>. Additionally, the co-localization of AnxA2 and Sc<Cn*MPR1*> suggest that successful crossing of the BBB is dependent on an AxnA2-Mpr1-mediated interaction. Collectively the data suggest that AnxA2 plays a central role in fungal transcytosis in human brain microvascular endothelial cells. The movement and exocytosis of Sc<Cn*MPR1*> is dependent on membrane trafficking events that involve AnxA2 but these events appear to be independent from the actions of AnxA2 at the host cell surface. We propose that Mpr1 activity promotes cytoskeleton remodeling in brain microvascular endothelial cells and thereby engages AnxA2 in order to facilitate fungal transcytosis of the BBB.

## Introduction

Infection of the central nervous system (CNS) causes significant morbidity and mortality. The mechanisms used by circulating eukaryotic pathogens to evade host immunity, cross the blood-brain barrier (BBB), and invade the CNS are remarkably complex and diverse (Ueno and Lodoen, 2015). Normally the brain microvascular endothelial cells that constitute the BBB prevent harmful substances from invading the CNS by restricting movement across the barrier; however, some microorgansims have evolved stealth-like mechanisms that allow them to breach the BBB. *Cryptococcus neoformans* (Cn) is an opportunistic fungal pathogen that causes a life-threatening infection of the brain most commonly in populations with impaired immunity (Bicanic and Harrison, 2004).

Cn can cross the BBB by a transcellular pathway and a Trojan horse (i.e., monocyte-assisted)-mediated mechanism (Charlier et al., 2009; Shi et al., 2010; Huang et al., 2011; Liu et al., 2013; Vu et al., 2013, 2014; Santiago-Tirado et al., 2017). At its core, transcytosis is simply the movement of a macromolecular payload across a cellular membrane (Tuma and Hubbard, 2003). The entry into cells involves endocytic mechanisms that include different modes of internalization. Among the key players that regulate the complexities of endocytosis, is Annexin A2—a calcium and phospholipid-binding protein that is often associated with the cell membrane and the cytoskeleton. (Grieve et al., 2012). Remodeling of the cytoskeleton via the plasma membrane-associated actin networks is central to endocytosis and macroendocytic events like macropinocytosis and phagocytosis. (Mercer and Helenius, 2009). In the case of *C. neoformans*, internalization by brain microvascular endothelial cells coincided with membrane ruffling and the formation of membrane protrusions suggesting a reorganization of the cytoskeleton (Chen et al., 2003; Chang et al., 2004). Further studies demonstrated that *C. neoformans* could induce actin cytoskeletal remodeling via the ROCK-LIM kinase-coffilin pathway (Chen et al., 2003).

The fungal components that directly target brain endothelial cells have yet to be fully elucidated, but some compelling evidence supports a central role for cell-surface and secreted proteins of Cn during CNS invasion. Extracellular phospholipase B (Santangelo et al., 2004; Chayakulkeeree et al., 2011), urease (Olszewski et al., 2004), laccase (Qiu et al., 2012), hyaluronic acid (Jong et al., 2012), and Mpr1 (Vu et al., 2014) all contribute to the ability of Cn to cause brain infection. The secreted fungal metalloprotease, Mpr1, was shown to be required for attachment and internalization of Cn by the BBB both *in vitro* and *in vivo* (Eigenheer et al., 2007; Vu et al., 2014). Mpr1 appeared to be specific for the BBB since Mpr1 was not required for dissemination or colonization of other organs like lungs, kidneys, spleen, or heart (Vu et al., 2014). Importantly, Mpr1 may be sufficient for transmigration because the sole expression of the *MPR1* gene from *C. neoformans* (Cn*MPR1*) into *Saccharomyces cerevisiae*, a yeast that cannot normally migrate across the BBB, suddenly gained the ability to do so (Vu et al., 2014). Mpr1 is an extracellular fungalysin metalloprotease that belongs to the M36 class of fungal-specific metalloproteases (Monod et al., 1993; Lilly et al., 2008; Fernandez et al., 2013; Li and Zhang, 2014). There is some evidence for their role in fungal pathogenesis but the protein targets of fungalysins including Mpr1 remain largely unresolved (Markaryan et al., 1994).

Based on our studies, we proposed that Mpr1 likely enhanced the permeability of the BBB by targeting and proteolytically altering surface proteins of brain endothelial cells (Vu et al., 2014). In order to identify and fully characterize BBB surface proteins that might be targeted by Mpr1 and function at the fungal-barrier interface, we exploited the gain of function that Mpr1 conferred onto *S. cerevisiae*. To this end, biotin-labeled surface proteins of human brain microvascular endothelial cells (HBMECs) were incubated either with a strain of Sc expressing Cn*MPR1* (Sc<Cn*MPR1*>) or an Sc-wild type (Sc-WT) strain. Isolated and eluted proteins were identified by LC-MS/MS and spectral counts determined the relative abundance of proteins. Approximately 62 proteins with a fold change of ≥2 were found to associate specifically with the Sc<Cn*MPR1*> strain. Several of the proteins identified were components of the actin cytoskeleton or associated proteins, including Annexin A2 (AnxA2). Given the central role of AnxA2 at the interface of actin dynamics and endocytosis, we examined the role of AnxA2 during the transcytosis of the Sc<Cn*MPR1*> strain. Through transcytosis assays, TEM analysis and immunofluorescence studies in an *in vitro* model of the human BBB, we found that inhibiting AnxA2 in HBMECs prevented the transcytosis of Sc<Cn*MPR1*> but unexpectedly it did not prevent the association or the internalization of Sc<Cn*MPR1*>. Collectively the data suggest that Mpr1 activity contributes to the F-actin cytoskeleton remodeling and promotes the transcytosis of Sc<Cn*MPR1*> by engaging AnxA2.

## Materials and methods

### Expression of the MPR1 gene from *C. neoformans* into *S. cerevisiae*

The cDNA of *MPR1* was isolated from a wild type strain of *C. neoformans* (MATα, serotypeA, var. *grubii*).(Vu et al., 2014) The cryptococcal *MPR1* (CnMPR1) cDNA was fused to a 6X-HIS tag at the C-terminus and cloned into the episomal yeast shuttle expression vector, p416 (ATCC# 87360; American Type Culture Collection, Manassas, VA, USA) according to standard methods. Plasmid p416 containing CnMPR1 was transformed into a W303 a wild type strain of *S. cerevisiae* (*ura3-1, can1-100, leu2-3, trp1-1, his3-11,15*). The transformed strain is referred to as Sc<Cn*MPR1*>.

### Reverse transcriptase PCR

ScWT and Sc<Cn*MPR1*> were cultured overnight at 30°C in yeast extract-peptone dextrose (YPD) and uracil dropout media respectively. The yeasts cells (~5 × 10^7^ cells) were harvested and lysed with acid-washed glass beads (425–600 μm acid-washed glass beads; Sigma-Aldrich, Saint Louis, MO, USA). RNA was isolated according to the manufacturer instruction (RNeasy Mini Kit; QIAGEN, Valencia, CA, USA), and cDNAs were synthesized using the first-strand cDNA kit (SuperScript III First-Strand Synthesis System; Invitrogen Life Technologies, Carlsbad, CA, USA). The cDNAs were used as templates for PCR reactions with the following primers: for Mpr1^6XHIS^, forward primer 5′ ATGCGCTCCTCCGCGCTCATC 3′ and reverse primer 5′ TCAATGGTGATGGTGATGATGTCTAGAAGATTTGG 3′; for actin (internal control), forward primer 5′ ATGGAAGAAGAAGTCGCCGCCTTGG 3′, and reverse primer 5′ TTAGAAACACTTTCGGTGGACGATTG 3′.

### Western blot analysis

Strains of *S. cerevisiae* (ScWT and Sc<Cn*MPR1*>) were lysed with Lysis Buffer (50 mM Tris-HCl pH 7.5, 150 mM NaCl, 5 mM NaF, 5 mM EDTA, 0.1% NP-40) supplemented with yeast protease inhibitors (Protease Inhibitor Cocktail P8215; Sigma-Aldrich, Saint Louis, MO, USA) and 1 mM DTT. The protein concentrations were measured by Bradford assay (Quick Start™ Bradford Protein Assay; Bio-Rad Laboratories Inc., Hercules, CA, USA), and the samples were denatured and reduced in Laemmli buffer (Premixed 4X Laemmli Protein Sample Buffer; Bio-Rad Laboratories Inc., Hercules, CA, USA) followed by boiling at 95°C for 3 min. SDS-PAGE electrophoresis was performed with 10% polyacrylamide gel at 90 V for 2 h (Mini-PROTEAN Tetra Cell; Bio-Rad Laboratories Inc., Hercules, CA, USA). The proteins on SDS-PAGE gel were transferred to a PVDF membrane (Immun-Blot PVDF membrane; Bio-Rad Laboratories Inc., Hercules, CA, USA) using the semi-dry transfer method (Trans-Blot SD Semi-Dry Transfer Cell; Bio-Rad Laboratories Inc., Hercules, CA, USA) at 15 V for 20 min. The PVDF membrane was stained with Ponceau Red (Ponceau S Stain; AMRESCO LLC, Solon, OH, USA) to visualize polypeptide bands, then blocked with 5% milk (Blotting-Grade Blocker; Bio-Rad Laboratories Inc., Hercules, CA, USA) and incubated with 1:1,000 dilution of the primary antibody (Mouse Anti-6X His Antibody Clone N144/14; UC Davis/NINDS/NIMH NeuroMab Facility, Davis, CA, USA) at 4°C overnight. The membrane was washed several times with TBST buffer and incubated with secondary antibody (1:5,000 dilution) (Goat Anti-Mouse IgG H&L HRP ab6789; Abcam Inc., Cambridge, MA, USA) at room temperature for 1 h. After washing with TBST, the chemiluminescent substrate solution for HRP (SuperSignal West Pico Chemiluminescent Substrate; Pierce Biotechnology, Rockford, IL, USA) was added to the membrane, and the X-ray films (CL-X Posure Film; Pierce Biotechnology, Rockford, IL, USA) were used to detect expression of Mpr1 protein.

### Immunofluorescence

To detect Mpr1 expression and localization in *S. cerevisiae*, ScWT and Sc<Cn*MPR1*> strains were grown in YPD and uracil dropout media respectively at 30°C overnight. The log-phase yeast cells (OD_600_ = ~1.0) were harvested and fixed in 2% paraformaldehyde at room temperature for 30 min. The fixed cells were washed twice with 0.1 M phosphate buffer pH 6.5 and *S. cerevisiae* cell walls were digested with 5% zymolase (Longlife Zymolase; G-Biosciences, Saint Louis, MO, USA) in 0.1 M phosphate buffer containing 0.5 M sorbitol and 1% β-mercaptoethanol at 37°C for 30 min. *S. cerevisiae* strains were washed twice with 0.1 M phosphate buffer containing 0.5 M sorbitol and incubated with 1:500 dilution of the 6X-HIS tag antibody at 4°C overnight. Subsequently, 1:500 dilution of secondary antibody (Goat Anti-Mouse IgG H&L Alexa Fluor 488 ab150117; Abcam Inc., Cambridge, MA, USA) was added, incubated at room temperature for 1 h, and eventually washed twice with 0.1 M phosphate buffer. The slides were mounted with aqueous mounting medium (VectaMount AQ; Vector Laboratories Inc., Burlingame, CA, USA) and imaged using a fluorescence microscope (Leica DMR series fluorescence microscope; Chroma Technology, Rockingham, VT, USA).

For immunofluorescence studies of hCMEC/D3 cells, the cells were grown in 12-transwells (Cell Culture Insert PET membrane 8.0 μm pore size; Corning Inc., Lowell, MA, USA) for ~2 weeks until the cells were differentiated (see the cell and culture condition methods). *S. cerevisiae* strains (ScWT and Sc<Cn*MPR1*>) were labeled with fluorescein isothiocyanate (FITC mixed isomer; Pierce Biotechnology, Rockford, IL, USA) and incubated with hCMEC/D3 at 37°C, 5% CO_2_ for 1.5 h. Cells were fixed in ice-cold methanol at −20°C for 20 min, subsequently washed with 100% acetone, permeabilized with 0.1% TritonX-100 for 10 min, and washed twice with Immunofluorescence Buffer (ImF buffer; 0.15 M NaCl, 5 mM EDTA, 20 mM HEPES pH 7.5). A 1:500 dilution of the Annexin A2 antibody (Anti-ANXA2 Monoclonal Antibody Clone 1G7; Abnova, Taipei, Taiwan) in 1% BSA was added to fixed cells and cells were incubated at 4°C overnight. After washing cells with ImF buffer three times, the secondary antibody (Goat Anti-Mouse IgG H&L Alexa Fluor 555 ab150114; Abcam Inc., Cambridge, MA, USA) at 1:1,000 dilution was added for 1 h at room temperature. DAPI (DAPI; Cell Signaling Technologies, Danvers, MA, USA) was used for staining nuclei of hCMEC/D3. The immunofluorescence images were obtained using a confocal microscope (Leica TCS SP8 STED 3X; Leica Microsystems Inc., Buffalo Grove, IL, USA).

### Scanning and transmission electron microscopy (SEM and TEM)

ScWT and Sc<Cn*MPR1*> strains were grown in YPD and uracil dropout media respectively at 30°C overnight. Strains were washed three times with PBS and fixed in modified Karnovsky's fixative (2% paraformaldehyde, 2.5% glutaraldehyde in 0.06 M Sorensen's phosphate buffer pH 7.3). For TEM, hCMEC/D3 were pretreated with the Annexin A2 antibody and infected with ScWT or Sc<Cn*MPR1*> yeast strains as described above. After 3-h incubation at 37°C, 5% CO_2_, the cell culture media were removed, and the hCMEC/D3 were fixed in modified Karnovsky's fixative and submitted to Electron Microscopy Core Laboratory at University of California, Davis for SEM TEM sample preparation. The SEM images were obtained using Philips XL30 TMP scanning electron microscope (F.E.I. Company, Hillsboro, OR, USA). TEM imaging obtained with TEM microscope (Phillips CM120 Biotwin Lens; F.E.I. Company, Hillsboro, OR, USA; with: Gatan MegaScan model 794/20 digital camera (2K X 2K), Gatan BioScan model 792, Pleasanton, CA, USA).

### Cell culture conditions

The hCMEC/D3 cell line (human brain microvascular endothelial cells) was provided by B. Weksler (Cornell University). The cells were used between passages 20 and 30. Endothelial cell growth medium supplemented with growth factors, antibiotics and 5% fetal bovine serum (EGM-2 BulletKit and EBM-2 Basal Medium; Lonza, Walkerville, MD, USA) was used for growing the cells until 90–95% confluent at 37°C and 5% CO_2_. Medium was then changed to a 1/2 dilution and to two 1/4 dilutions, respectively, every 3–4 days in order to reduce growth factors and promote differentiation of hCMEC/D3 cells to ensure a tight-barrier formation. For biotinylation of brain endothelial cell surface proteins, hCMEC/D3 cells were cultured in 75 cm^2^ cell culture flasks coated with collagen (Nunc Cell Culture Treated EasYFlasks; Thermo Fisher Scientific, Nalge Nunc International, Rochester, NY, USA) (Collagen I, Rat Tail; Corning, Discovery Labware Inc., Bedford, MA, USA). For the immunofluorescence of hCMEC/D3, the association assay, the transcytosis assay, the FITC-dextran permeability, and the transmission electron microscopy (TEM), hCMEC/D3 were grown in collagen-coated transwells (see the methods below).

### Biotinylation of brain endothelial cell surface proteins

Cell surface proteins of hCMEC/D3 were biotinylated according to the manufacturer's instructions (Pierce Cell Surface Protein Isolation Kit; Pierce Biotechnology, Rockford, IL, USA). Briefly, hCMEC/D3 were washed twice with ice-cold PBS and labeled with sulfo-NHS-SS-biotin at 4°C for 30 min. Cells were collected, washed with TBS and lysed in IP Lysis Buffer (Pierce™ Direct Magnetic IP/Co-IP Kit; Pierce Biotechnology, Rockford, IL, USA) with protease inhibitors (Halt™ Protease Inhibitor Single-Use Cocktail; Pierce Biotechnology, Rockford, IL, USA) by incubating on ice for 30 min with gentle vortexing every 10 min. The cell lysate was centrifuged at 13,000 rpm for 20 min. The supernatant was collected and protein concentration was measured by bicinchoninic acid (BCA) protein assay (Pierce BCA Protein Assay Kit; Pierce Biotechnology, Rockford, IL, USA). The biotinylated cell surface proteins were isolated via magnetic beads (Pierce™ Direct Magnetic IP/Co-IP Kit; Pierce Biotechnology, Rockford, IL, USA) coupled to anti-biotin antibody (Rabbit Anti-Biotin Antibody ab53494; Abcam Inc., Cambridge, MA, USA). The proteins eluted from the magnetic beads were quantified with Bradford protein assay (Quick Star™ Bradford Protein Assay; Bio-Rad Laboratories Inc., Hercules, CA, USA).

### Identification of brain endothelial cell surface proteins targeted Mpr1

ScWT and Sc<Cn*MPR1*> were cultured in YPD and uracil dropout media respectively at 30°C overnight. The yeast cells were washed twice with PBS-CM (1X PBS, 0.9 mM CaCl_2_, 0.49 mM MgCl_2_) and resuspended in PBS-CM with 0.75% w/v n-octyl-β-D-glucopyranoside (O8001; Sigma-Aldrich, Saint Louis, MO, USA). 5 × 10^8^ cells of Sc<CnMPR1> (and ScWT as a control group) were incubated with 200 μg of isolated hCMEC/D3 cell surface proteins at 4°C for 1 h. Yeast cells were washed with ice-cold PBS-CM supplemented with 0.75% w/v n-octyl-β-D-glucopyranoside 3x to remove unbound proteins, and hCMEC/D3 cell surface proteins remaining attached to yeast cells were eluted with Elution buffer (4.5 M Urea, 1 M sorbitol, 200 mM NaCl, 10 mM EDTA, 0.1 M sodium phosphate buffer pH 7.4) for 5 min.

The eluted proteins from both the Mpr1-expresssing group (Sc<Cn*MPR1*>) and control group (ScWT) were sent to the Proteomics Core Facility at UC Davis for protein identification using sensitive liquid chromatography tandem mass spectrometry (LC-MS/MS). The analysis was performed as previously described (Vu et al., 2013). Briefly, X!tandem was used for database searching, and the proteins specifically bound to Sc<Cn*MPR1*> were identified with a protein threshold >99.0% and ≥ 3 unique peptides of <0.1 false discovery rate (FDR) using Scaffold 4. From 1,348 total proteins, 62 human proteins were selected and categorized based on GO terms associated with known or predicted biological function.

### Association assay

The *in vitro* model of the human BBB used here was previously described. (Weksler et al., 2005; Vu et al., 2009, 2014) Briefly hCMEC/D3 were grown on inserts of collagen-coated 24-transwells (Cell Culture Insert PET membrane 8.0 μm pore size; Corning Inc., Lowell, MA, USA) until the cells differentiated into a tight barrier. hCMEC/D3 cells were washed twice with PBS and pretreated with 2.3 μg of Annexin A2 antibody at 37°C, 5% CO_2_ for 40 min. 6.9 × 10^4^ cells of ScWT or Sc<Cn*MPR1*> strains were added into each transwell. Following a 1-h incubation at 37°C and 5% CO_2_, hCMEC/D3 cells were washed 5X with PBS. In order to collect the *S. cerevisiae* cells that specifically associated with hCMEC/D3, water was added in order to rupture the hCMEC/D3 cells. The contents was collected and plated onto YPD agar plates. Following incubation at 30°C for 3 days, CFUs were determined.

### Transcytosis and FITC-dextran permeability assays

hCMEC/D3 were grown in 96-transwells of the *in vitro* model of the BBB (HTS Transwell-96 Well Plate PET membrane 8.0 μm pore size; Corning Inc., Lowell, MA, USA) as described previously. (Weksler et al., 2005; Vu et al., 2009, 2014) One microgram of the annexin A2 antibody or the control antibody (Mouse IgG1 Monoclonal NCG01 Isotype Control; Abcam Inc., Cambridge, MA, USA) was added to each upper chamber and incubated at 37°C, 5% CO_2_ for 40 min. hCMEC/D3 cells were infected with ~3 × 10^4^ cells of ScWT or Sc<Cn*MPR1*> strains at 37°C, 5% CO_2_. At 6-h post-infection, the media in the lower chambers was collected and plated onto YPD agar plates to quantify the number of *S. cereviaise* cells that had crossed the BBB.

To determine the integrity of the *in vitro* BBB model, FITC-dextran permeability assay was performed in parallel with the transcytosis assays. At time point 0 of the transcytosis, a 1 μg/μl final concentration of FITC- conjugated dextran (Fluorescein Isothiocyanate-dextran mol wt 70,000; Sigma-Aldrich, Saint Louis, MO, USA) was added into the upper chambers of each transwell. Following a 6-h post-incubation, fluorescence intensity of the media in the upper and the lower chambers was quantified at 538 nm (excitation of 485 nm) using a microplate reader (SpectraMax M5; Molecular Devices, Sunnyvale, CA, USA) with SoftMax Pro 5.2 software.

### Statistical analysis

Statistical significance was determined by running one-way analysis of variance (ANOVA) and *T*-test using GraphPad Prism Program (GraphPad Software Inc.). *P*-values < 0.05 were considered significant.

## Results

### *Saccharomyces cerevisiae* migrated across brain microvascular endothelial cells (hBMECs) upon expression of Cn*MPR1*

**Figure 1:**
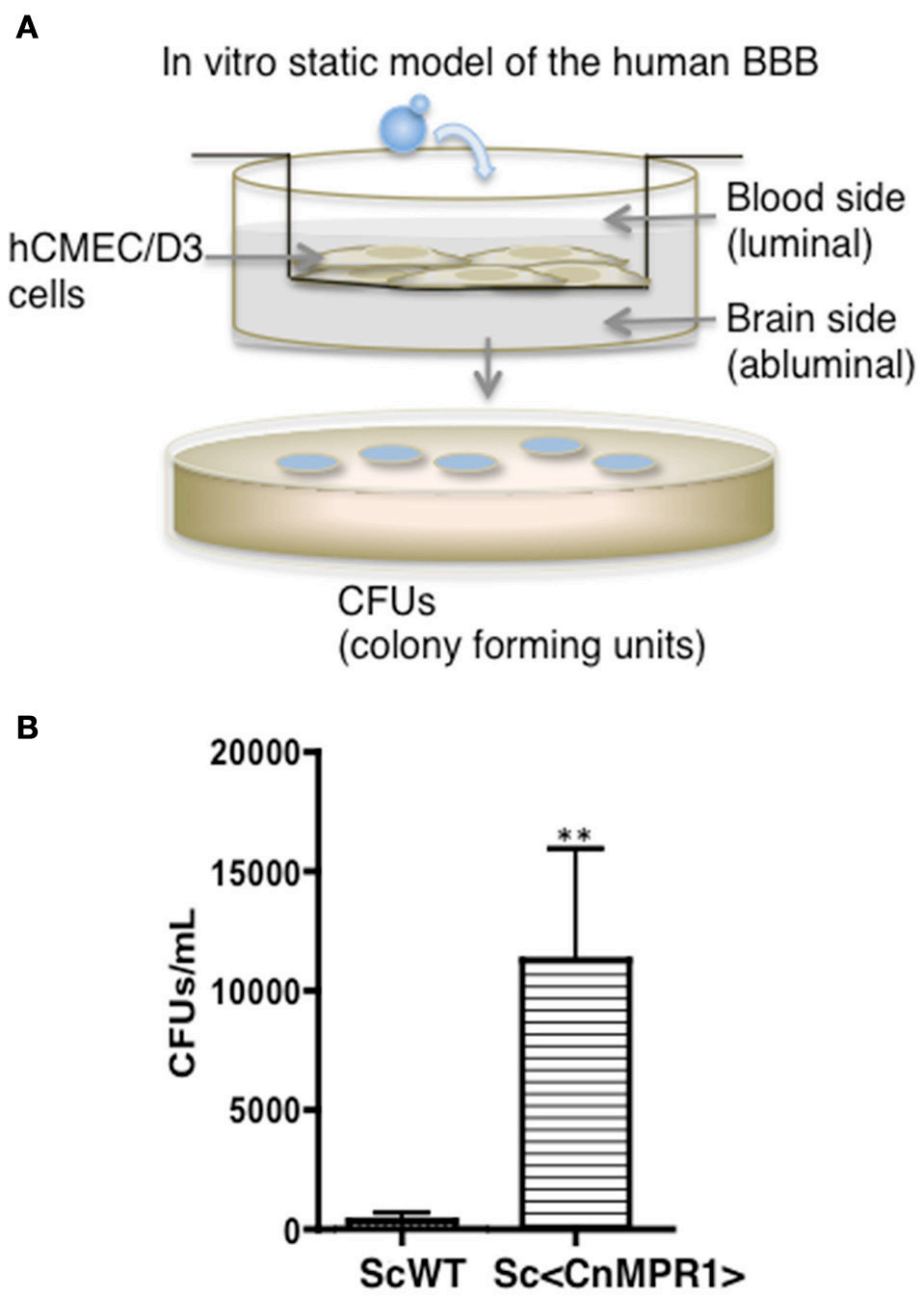
*Saccharomyces cerevisiae (Sc)* expressing cryptococcal *MPR1* (Sc<Cn*MPR1*>) gains ability to cross the BBB in *in vitro* model. **(A)** A schematic diagram of the static model of the human BBB. An immortalized human brain microvascular endothelial cell line (hCMEC/D3) was grown on collagen-coated transwell inserts submerged in cell culture medium such that the top chamber represented the blood (luminal) side and the bottom chamber represented the brain (abluminal) side. Yeast cells (*S. cerevisiae*) were added to the luminal side and incubated at 37°C with 5% CO_2_. Following a 6-h co-incubation, the media of the abluminal side (containing Sc that crossed the BBB) were collected and plated on YPD agar for colony counting. **(B)** Transcytosis assays in the human BBB model revealed that a strain of Sc expressing Cn*MPR1* cDNA (Sc<Cn*MPR1*>) crossed the BBB as shown by higher CFU counts compared to ScWT (*P* < *0.05, n* = 6). ^**^Indicates very significant with *P* < 0.01.

The aim of this study was to resolve the role of Mpr1 in promoting a permeable BBB by exploiting the crossing ability conferred onto *S. cerevisiae* (Sc) when expressing Cn*MPR1* cDNA. Implementing an *in vitro* model of the human BBB (Figure 1A) (Weksler et al., 2005; Vu et al., 2009, 2014), we demonstrated through transcytosis assays that *S. cerevisiae* gained the ability to cross human brain microvascular endothelial cells (hBMECs) upon the expression of CnMPR1 cDNA in Sc (referred to as, Sc<Cn*MPR1*> strain) (Figure 1B) (Vu et al., 2014). We have confirmed that recombinant Cn*MPR1* isolated from the Sc<Cn*MPR1*> strain maintained proteolytic activity, and this activity was required for crossing the BBB (data not shown) (Vu et al., 2014).

**Figure 2:**
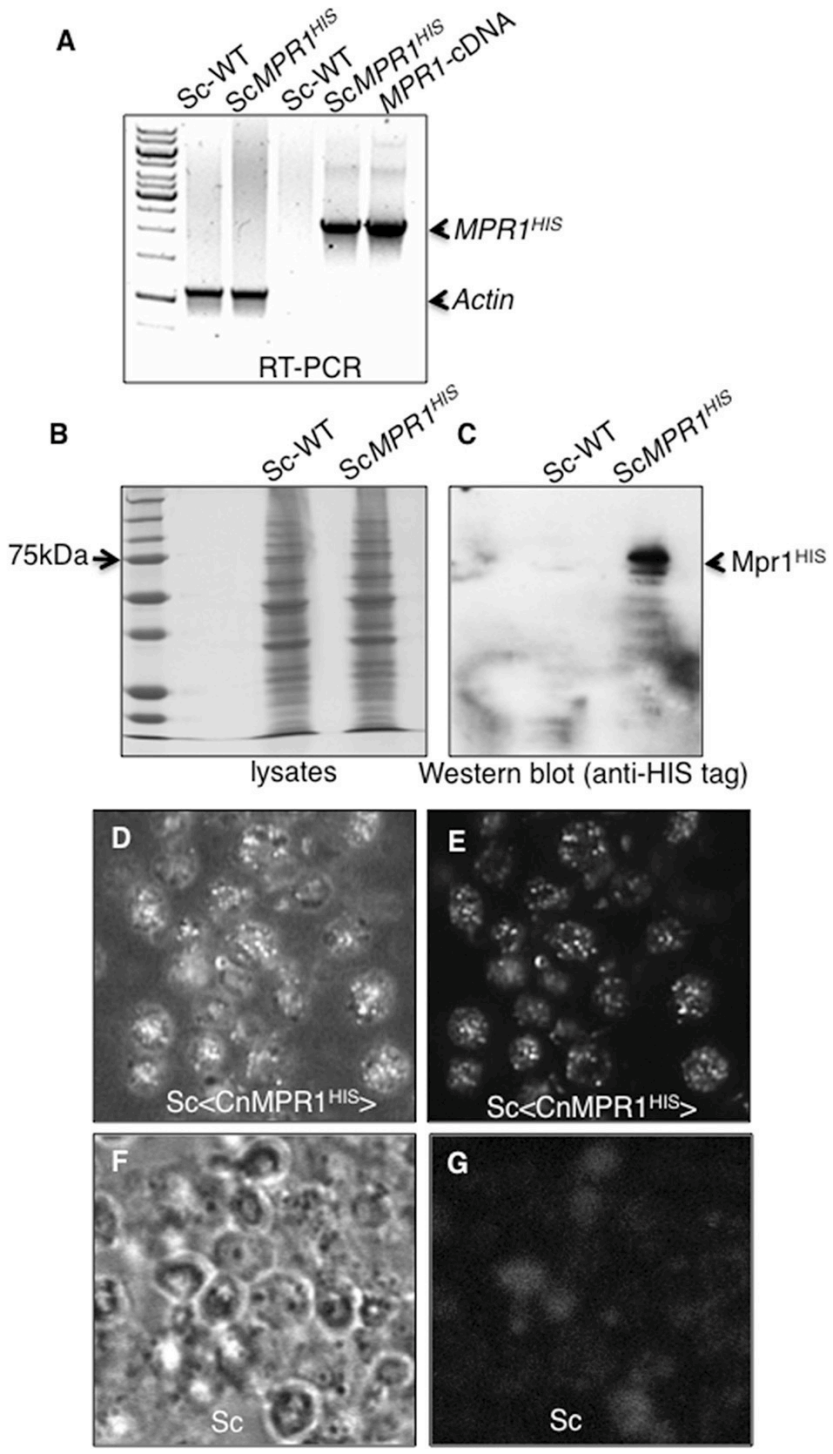
Expression of Cn*MPR1-HIS* tagged cDNA in *S. cerevisiae*. **(A)** Reverse transcriptase-PCR (RT-PCR) detected mRNA transcript levels of Cn*MPR1* in Sc. An Sc strain was transformed with a plasmid containing a C-terminal HIS-tagged *MPR1* cDNA isolated from *C. neoformans* (Sc<CnMPR1^*HIS*^>). Transcripts of *MPR1* were not detected in a wild type strain of Sc (ScWT). The plasmid containing Cn*MPR1-*cDNA, used as a positive control for PCR, indicated a cDNA size of ~2.1 kb similar to that detected for Sc<*CnMPR1*^*HIS*^>. β-actin was used as an internal control for RT-PCR. **(B,C)** Western blot analysis of Mpr1^*HIS*^ in whole cell lysates of ScWT and Sc<*CnMPR1*^*HIS*^> revealed expression of Mpr1 protein in Sc<*CnMPR1*^*HIS*^>. **(D–G)** Indirect immunofluorescence further confirmed the expression of Mpr1 in Sc<*CnMPR1*^*HIS*^>. Bright field images **(D,F)** and corresponding fluorescence images **(E,G)** are shown. **(E)** Mpr1^*His*^ was detected with a 6X-HIS primary antibody followed by a secondary antibody (Alexa Fluor 488) in Sc<*CnMPR1*^*HIS*^>. **(G)** Mpr1 protein was not detected in ScWT.

The expression of CnMPR1 in Sc was accomplished by subcloning the HIS-tagged cDNA of Cn*MPR1* adjacent to a constitutive promoter in a yeast expression vector. Reverse transcriptase PCR confirmed the expression of the *MPR1* mRNA transcript only in strains of Sc transformed with Cn*MPR1*^*HIS*^-plasmid DNA and not in a wild type strain of Sc (Figure 2A). The mRNA transcript of Cn*MPR1*^*HIS*^ in Sc detected by RT-PCR was similar in size to the cDNA-*MPR1* plasmid control (Figure 2A). Sc expressing Cn*MPR1*^*HIS*^ was cultured and lysed and isolated polypeptides were separated by protein gel electrophoresis and analyzed by Western blot analysis (Figures 2B,C). An anti-HIS tag antibody detected a prominent band at ~70 kDa unique to Sc cells expressing Cn*MPR1*^*HIS*^ (Figure 2C).

We next examined the localization of Cn*MPR1* in *S. cerevisiae* by immunofluorescence. Sc expressing Cn*MPR1*^*HIS*^ were grown to mid-log-phase, fixed and exposed to a HIS-tag primary antibody followed by an Alexa488 secondary antibody. Fluorescence microscopy revealed a punctate pattern for Cn*MPR1* in Sc (Figures 2D,E) that was not detected in wild type Sc (Figures 2F,G). The localization pattern observed for Mpr1 was typical of secreted proteins. Thus, the secretion of Cn*MPR1* in yeast and the gain of function conferred onto Sc by Cn*MPR1* prompted us to ask whether Mpr1 activity promoted the internalization of Sc<Cn*MPR1*> by altering the Sc cell surface.

### Expression of Cn*MPR1* in Sc does not alter the surface architecture of Sc

**Figure 3:**
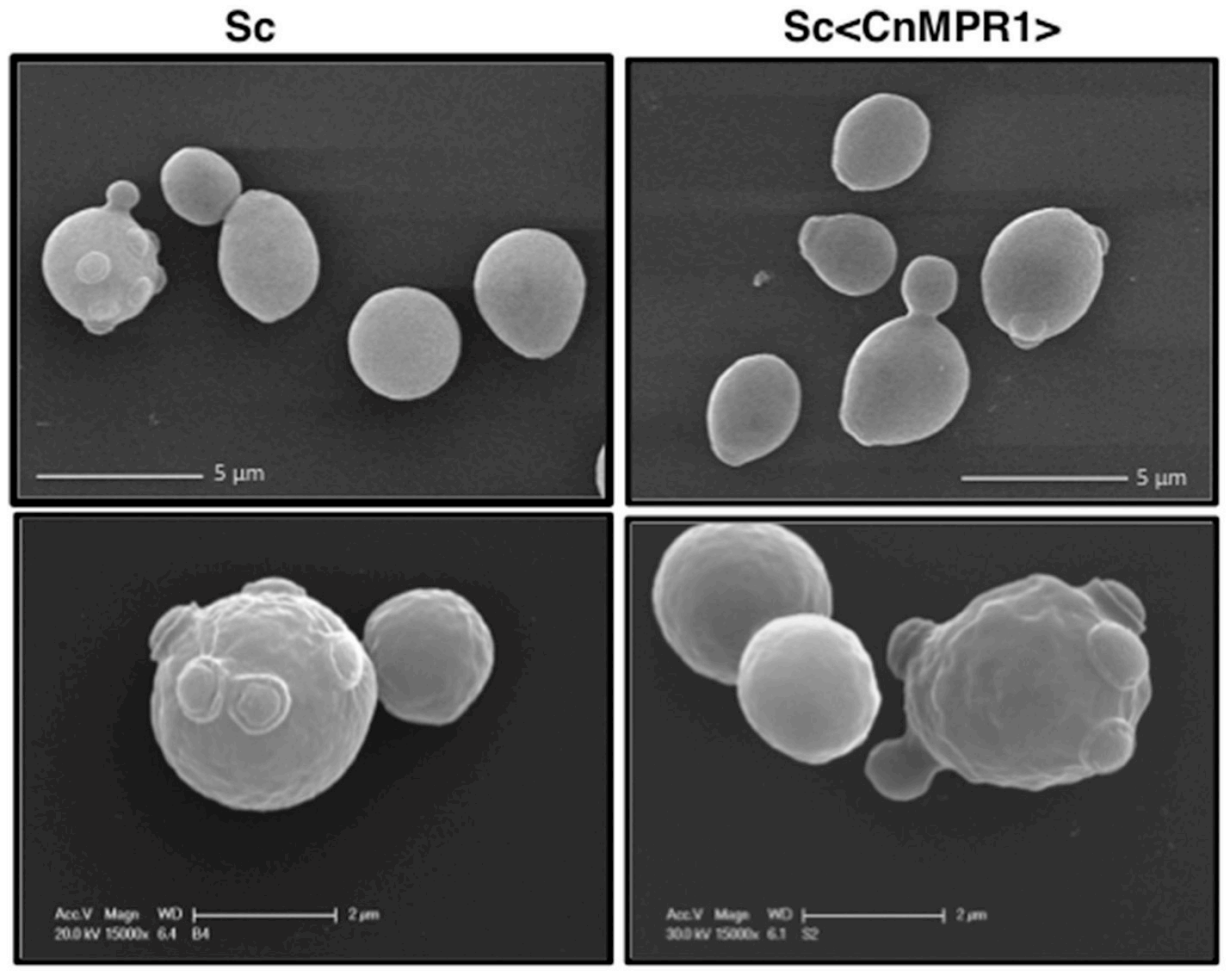
SEM imaging reveals no morphological changes of the yeast surfaces following expression of Cn*MPR1* cDNA in *S. cerevisiae*. ScWT (**Left**) and Sc<*CnMPR1*> (**Right**) were grown to mid-log phase, fixed, and their cell surfaces were observed with SEM. The SEM micrographs revealed similar size, shape, surface architecture (**Top**, **Bottom**), and morphology of bud scars (**Bottom**) between ScWT and Sc<Cn*MPR1*> strains.

Electron micrographs from scanning electron microscopy (SEM) of Sc expressing Cn*MPR1*^*HIS*^ revealed no obvious changes on the surface of Sc suggesting that Mpr1 activity may not have altered the extracellular architecture of Sc (Figure 3). SEM analysis revealed similar size, shape, surface morphology, and the presence of bud scars between ScWT and Sc expressing Cn*MPR1*^*HIS*^ (Figure 3). In addition, we did not detect any differences in growth or other discernable phenotypes (data not shown). These observations along with our previous published studies of fungal-BBB interactions, led to a working hypothesis that Mpr1 conferred BBB-crossing ability to Sc by targeting proteins on the surface of hBMECs (Eigenheer et al., 2007; Vu et al., 2009, 2013, 2014).

### The Sc<Cn*MPR1*> strain targets proteins on the surface of hBMECs

**Figure 4:**
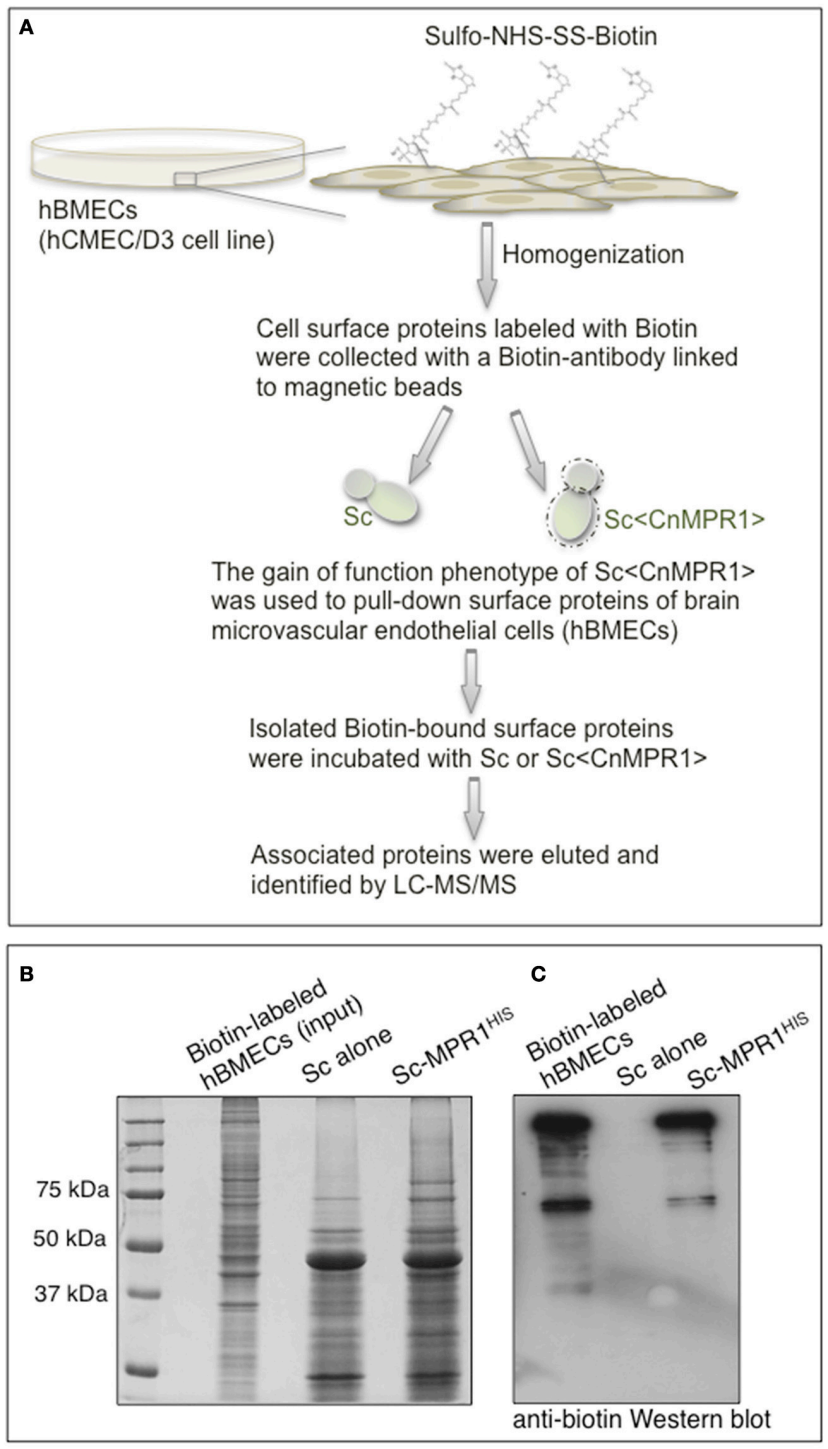
*S. cerevisiae* expressing Cn*MPR1* (Sc<Cn*MPR1*>) binds to cell surface proteins of hBMECs. **(A)** A work flow diagram representing the methods used for identifying host targets of Mpr1. Cell surface proteins of hBMECs (hCMEC/D3 cell line) were labeled with Sulfo-NHS-SS-Biotin and isolated via magnetic beads coupled to a biotin antibody. Intact cells of Sc<Cn*MPR1*> were co-incubated with the isolated hBMECs surface proteins for 1 h at 4°C; Sc<Cn*MPR1*> cells were used to pull down Mpr1-associated host proteins. ScWT was used as a negative control for this assay. After the co-incubation, unbound proteins were removed by extensive washing, while bound proteins were eluted and subjected to tandem mass spectrometry for protein identification. **(B)** The total biotin-labeled hBMECs, and the ScWT- and Sc<*CnMPR1*>-eluted proteins were observed by gel electrophoresis showing that among the total protein input only a fraction of proteins was eluted after co-incubation with Sc<*CnMPR1*>. **(C)** An anti-biotin Western blot analysis of biotin-labeled hBMECs proteins and eluted proteins revealed distinct bands associated with Sc<CnMPR1>, suggesting that surface proteins of hBMECs associated more readily with Sc expressing Cn*MPR1*.

In order to identify surface proteins of hBMECs that were likely mediating interactions at the yeast-BBB interface, we co-incubated isolated biotin-labeled surface proteins of hBMECs with intact cells of either Sc-wild type strain or Sc<Cn*MPR1*> strain (Figure 4A). The sulfo-NHS-SS-Biotin used here was a thiol-cleavable amine-reactive biotinylation. This compound was particularly useful for labeling cell surface proteins since its sulfonate group prevented it from permeating cell membranes. The bound proteins were washed extensively, eluted and examined by gel electrophoresis and Western blot (Figure 4B). Protein gel analysis revealed that of the total number of biotin-labeled proteins (i.e., hBMECs, Input) only a fraction was eluted following incubation with Sc strains (Figure 4B). Western blot analysis showed that several of these polypeptides appeared to be unique to the yeast strain expressing Cn*MPR1* (Figure 4C).

The eluted proteins were identified by LC-MS/MS via spectral analysis and grouped according to known or predicted function (Table 1) (Vu et al., 2013). We identified 62 proteins with at least a two-fold change in expression in hBMECs exposed to Sc<Cn*MPR1*> compared to hBMECs exposed to ScWT alone (Table 1). The largest cluster of proteins associated with Sc<Cn*MPR1*> were proteins involved in membrane rearrangement and cytoskeleton remodeling. Filamin, profilin, a Ras GTPase-activating protein (IQGAP1) and annexinA2 (AnxA2) were among the proteins with the largest fold-change (Table 1). These results are consistent with a previous study where we demonstrated a similar protein profile of hBMECs exposed to a strain of *C. neoformans* (Vu et al., 2013). In addition several studies have suggested a key role for the cytoskeleton during pathogen penetration of the BBB thus further supporting the protein profile identified in this study (Chen et al., 2003; Eigenheer et al., 2007; Huang et al., 2011).

**Table 1:** Identified proteins from hBMECs targeted by Mpr1 grouped according to biological function.

**Protein name**	**Accession number**	**Molecular weight (kDa)**	**Sc <CnMpr1> + hCMEC/D3**	**ScWT + hCMECD3**	**Fold change**
**CYTOSKELETON AND MEMBRANE REARRANGEMENT**					
Cluster of Myosin-9	sp|P35579|MYH9_HUMAN	227	6,602	1,301	6.9
Cluster of Actin, cytoplasmic 2	sp|P63261|ACTG_HUMAN	42	667	230	3.9
Cluster of Tubulin beta chain	sp|P07437|TBB5_HUMAN	50	269	74	4.9
Cluster of Tubulin alpha-1B chain	sp|P68363|TBA1B_HUMAN	50	143	33	5.8
Cluster of Myosin light polypeptide 6	tr|G8JLA2|G8JLA2_HUMAN	17	127	25	6.6
Cluster of Tight junction protein ZO-2	sp|Q9UDY2|ZO2_HUMAN	134	122	7	24
Cluster of Talin-2	sp|Q9Y4G6|TLN2_HUMAN	272	86	4	29
Cluster of Unconventional myosin-Ic	sp|O00159|MYO1C_HUMAN	122	82	6	19
Cluster of Alpha-actinin-1	sp|P12814|ACTN1_HUMAN	103	30	17	2.5
Cluster of Moesin	sp|P26038|MOES_HUMAN	68	61	9	9.6
Tight junction protein 1 (Zona occludens 1), isoform CRA_a	tr|G3V1L9|G3V1L9_HUMAN	197	63	4	21
Cluster of Filamin-A	sp|P21333|FLNA_HUMAN	281	53	1	36
Cluster of Annexin A2	sp|P07355|ANXA2_HUMAN	39	46	4	16
Cluster of Unconventional myosin-Ib	sp|O43795|MYO1B_HUMAN	132	42	2	28
Cluster of Myosin regulatory light chain 12B	sp|O14950|ML12B_HUMAN	20	38	12	3.9
Heat shock protein beta-1	sp|P04792|HSPB1_HUMAN	23	26	1	35
Profilin-1	sp|P07737|PROF1_HUMAN	15	36	1	49
Cluster of F-actin-capping protein subunit beta	sp|P47756|CAPZB_HUMAN	31	29	2	20
Cluster of Cofilin-1	sp|P23528|COF1_HUMAN	19	23	4	7.8
Cluster of F-actin-capping protein subunit alpha-1	sp|P52907|CAZA1_HUMAN	33	28	2	19
Ras GTPase-activating-like protein IQGAP1	sp|P46940|IQGA1_HUMAN	189	20	1	32
Cluster of Actin-related protein 3	sp|P61158|ARP3_HUMAN	47	14	1	19
**CELLULAR TRANSPORTATION**					
Cluster of Unconventional myosin-Ic	sp|O00159|MYO1C_HUMAN	122	82	6	19
Major vault protein	sp|Q14764|MVP_HUMAN	99	66	2	45
Cluster of Endoplasmin	sp|P14625|ENPL_HUMAN	92	51	8	6.2
Cluster of Unconventional myosin-Ib	sp|O43795|MYO1B_HUMAN	132	42	2	28
Transitional endoplasmic reticulum ATPase	sp|P55072|TERA_HUMAN	89	37	2	18
Cluster of Clathrin heavy chain 1	sp|Q00610|CLH1_HUMAN	192	25	2	17
**METABOLIC PROCESS**					
Cluster of Pyruvate kinase PKM	sp|P14618|KPYM_HUMAN	58	96	17	7.6
Alpha-enolase	sp|P06733|ENOA_HUMAN	47	90	15	7.6
Cluster of Fructose-bisphosphate aldolase A	sp|P04075|ALDOA_HUMAN	39	68	6	16
Cluster of Transketolase	sp|P29401|TKT_HUMAN	68	58	6	13
Cluster of L-lactate dehydrogenase A chain	sp|P00338|LDHA_HUMAN	37	44	5	12
Neutral alpha-glucosidase AB	sp|Q14697|GANAB_HUMAN	107	33	1	45
Triosephosphate isomerase	sp|P60174|TPIS_HUMAN	31	26	4	8.8
Cluster of ATP-dependent 6-phosphofructokinase, platelet type	sp|Q01813|PFKAP_HUMAN	86	28	1	39
Phosphoglycerate mutase 1	sp|P18669|PGAM1_HUMAN	29	20	3	9
**NUCLEOPROTEINS AND RIBONUCLEOPROTEIN AND PROTEIN SYNTHESIS**					
Cluster of Nucleolin	sp|P19338|NUCL_HUMAN	77	163	15	15
Nucleophosmin	sp|P06748|NPM_HUMAN	33	53	4	12
Elongation factor 2	sp|P13639|EF2_HUMAN	95	51	5	2.8
Heterogeneous nuclear ribonucleoprotein U	sp|Q00839|HNRPU_HUMAN	91	39	2	26
Heterogeneous nuclear ribonucleoprotein A1	sp|P09651|ROA1_HUMAN	39	48	6	11
Cluster of Heterogeneous nuclear ribonucleoprotein Q	sp|O60506|HNRPQ_HUMAN	70	23	1	31
Heterogeneous nuclear ribonucleoproteins A2/B1	sp|P22626|ROA2_HUMAN	37	22	2	15
Splicing factor U2AF 65 kDa subunit	sp|P26368|U2AF2_HUMAN	54	15	4	5.4
Heterogeneous nuclear ribonucleoprotein K	sp|P61978|HNRPK_HUMAN	51	18	1	24
Cleavage and polyadenylation specificity factor subunit 6	sp|Q16630|CPSF6_HUMAN	59	18	2	12
**IMMUNITY**					
Cluster of Interferon-induced GTP-binding protein Mx2	sp|P20592|MX2_HUMAN	82	124	10	17
Cluster of HLA class I histocompatibility antigen, A-11 alpha chain	sp|P13746|1A11_HUMAN	41	44	3	20
E3 ubiquitin-protein ligase TRIM21	sp|P19474|RO52_HUMAN	54	39	3	12
Sequestosome-1	sp|Q13501|SQSTM_HUMAN	48	27	1	36
**PROTEIN FOLDING**					
Cluster of Protein disulfide-isomerase A3	sp|P30101|PDIA3_HUMAN	57	40	10	5.4
78 kDa glucose-regulated protein	sp|P11021|GRP78_HUMAN	72	48	3	6.9
Peptidyl-prolyl cis-trans isomerase A	sp|P62937|PPIA_HUMAN	18	39	8	6.6
Protein disulfide-isomerase A6	sp|Q15084|PDIA6_HUMAN	48	17	4	5.7
Cluster of Protein disulfide-isomerase	sp|P07237|PDIA1_HUMAN	57	14	1	19
**CELL SIGNALING**					
Cluster of Heat shock protein HSP 90-beta	sp|P08238|HS90B_HUMAN	83	90	9	7.8
Major vault protein	sp|Q14764|MVP_HUMAN	99	66	2	45
Calreticulin	sp|P27797|CALR_HUMAN	48	35	11	4.8
Cluster of Adenylyl cyclase-associated protein 1	sp|Q01518|CAP1_HUMAN	52	16	1	22
**DNA REPAIR**					
Transitional endoplasmic reticulum ATPase	sp|P55072|TERA_HUMAN	89	37	2	18
Ubiquitin-like modifier-activating enzyme 1	sp|P22314|UBA1_HUMAN	118	17	1	8.5

*Proteins were identified with a protein threshold >99.0% and ≥ 3 unique peptides of < 0.1 false discovery rate (FDR) using Scaffold 4. Proteins were categorized based on GO terms! associated with biological function and literature searches*.

### Association of Sc<Cn*MPR1*> with hBMECs is independent of AnxA2

**Figure 5:**
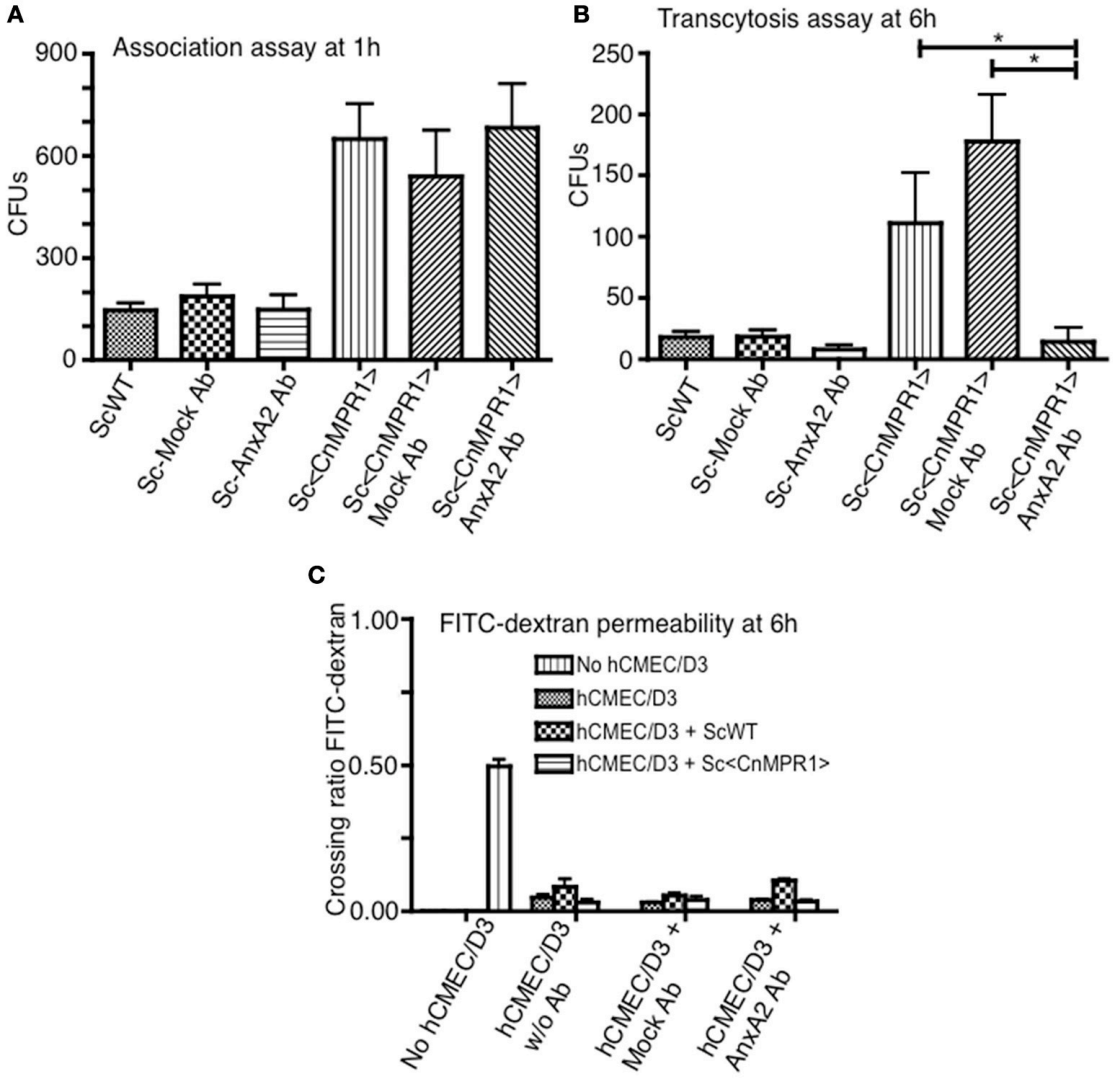
Inhibition of Annexin A2 (AnxA2) does not prevent the association of Sc<Cn*MPR1*> with hBMECs but reduces the transcytosis of Sc<Cn*MPR1*>. The *in vitro* models of BBB were used to investigate the association **(A)** and the transmigration of Sc<Cn*MPR1*> across the BBB **(B)**. The hBMECs were pretreated with the anti-AnxA2 or the IgG control antibody (mock treatment) for 40 min and subsequently incubated with ScWT or Sc<Cn*MPR1*> at 37°C with 5% CO_2_. Non-pretreated hBMECs were used as a control for both assays. **(A)** At 1 h post-co-incubation, hBMECs were extensively washed to remove unattached Sc, lysed, and plated on YPD agar for CFU counting; CFUs corresponded to the number of Sc associated with hBMECs. Blocking AnxA2 activity with an anti-AnxA2 antibody did not affect the association of Sc<Cn*MPR1*> with hBMECs (*P* > 0.05, *n* = 8). **(B)** At 6 h post-co-incubation transcytosis assays were performed where the cell culture media in the abluminal chambers of the *in vitro* BBB model was collected and plated on YPD agar. The CFU count showed a significant reduction of Sc<Cn*MPR1*> transmigration across the BBB in the presence of anti-AnxA2 antibody compared to no antibody and mock antibody (*P* < 0.05, *n* = 8). **(C)** The integrity of the barrier was monitored by measuring FITC-dextran permeability across hBMECs (fluorescent intensity of the abluminal chambers/luminal chambers). The low permeability ratios confirmed that the barrier remained intact throughout the assays (*P* > 0.05, *n* = 8). ^*^Indicates significant with *P* < 0.05.

We sought to further explore whether AnxA2 played a direct role in the BBB crossing of Sc<Cn*MPR1*>. To test this, the activity of AnxA2 was blocked by a monoclonal antibody against AnxA2 (AnxA2-Ab) in the *in vitro* model of the BBB. Surprisingly, blocking AnxA2 in hBMECs did not prevent Sc<Cn*MPR1*> from associating with hBMECs following a 1 h co-incubation (Figure 5A). The association assay revealed no significant difference in the CFUs of the Sc<Cn*MPR1*> strain following treatment of hBMECs with AnxA2-Ab, control antibody (IgG, mock Ab), or no antibody (Figure 5A). In striking contrast, the Sc<Cn*MPR1*> strain could no longer transcytose (i.e., cross) hBMECs treated with AnxA2-Ab suggesting either that the internalization or transcytosis of Sc<Cn*MPR1*> cells was blocked (Figure 5B). The CFUs of the Sc<Cn*MPR1*> strain were significantly different from hBMECs treated with AnxA2-Ab compared to control antibody (mock Ab) indicating that off-target effects did not play a role (Figure 5B). Permeability ratios of FITC-Dextran (70 kDa) revealed that blocking AnxA2 with AnxA2-Ab did not alter the tight junctions suggesting that the integrity of the barrier was intact during the course of the experiments and that the Sc<Cn*MPR1*> strain migrated via a transcellular route (Figure 5C). Collectively the assays in the *in vitro* model of the BBB demonstrated that AnxA2 was not required for initial association of Sc<Cn*MPR1*> with hBMECs but appeared to play a central role in mediating the crossing or transcytosis of Sc<Cn*MPR1*> through hBMECs (i.e., BBB). Based on these results, we concluded that AnxA2 is likely required during engulfing-internalization of Sc<Cn*MPR1*> or possibly the transcytosis and exit of Sc<Cn*MPR1*> from hBMECs.

### hBMECs lacking AnxA2 activity internalize Sc<Cn*MPR1*> but likely prevent its exit

**Figure 6:**
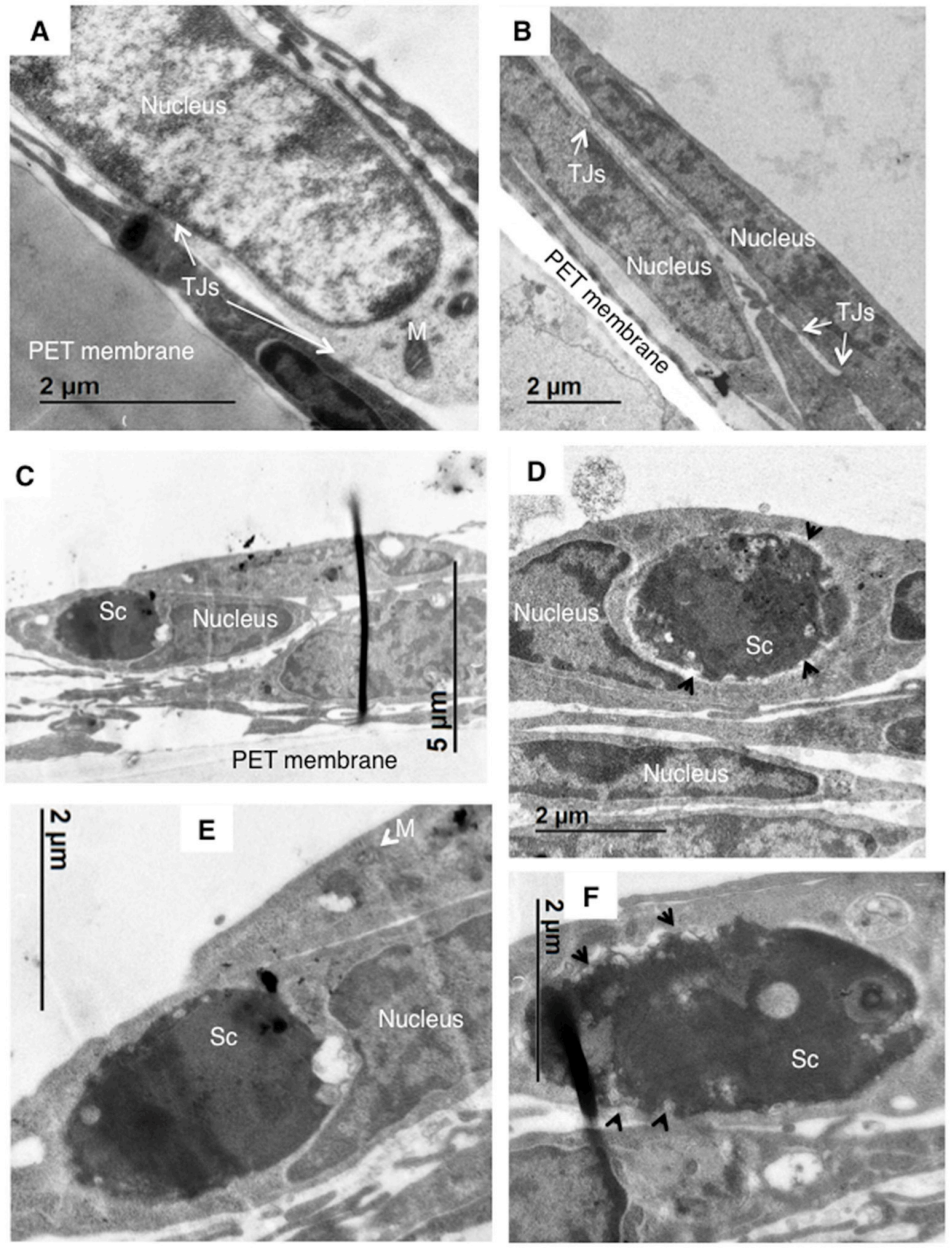
TEM imaging reveals that blocking AnxA2 activity in hBMECs does not affect the internalization Sc<Cn*MPR1*> but prevents the exit of of Sc<Cn*MPR1*> from hBMECs. hBMECs were grown on transwells in the *in vitro* model of BBB as described previously. **(A,B)** TEM images reveal the cellular structure of hBMECs, including a large nucleus, mitochondria (M, white arrows), and tight junctions (TJs) between the adjacent cells. **(C–F)** The activity of AnxA2 in hBMECs was blocked by pretreatment with anti-AnxA2 antibody followed by co-incubated with Sc<Cn*MPR1*> for 3 h at 37°C with 5% CO_2_. hBMECs were subsequently fixed, and prepared for TEM. The TEM images confirmed engulfment of Sc<Cn*MPR1*> (labeled as Sc) by hBMECs. Magnified images revealed a deteriorated Sc<Cn*MPR1*> cell surface (black arrows) suggesting that Sc<Cn*MPR1*> cells may be trapped within hBMECs due to the inhibition of AnxA2 activity and likely damaged by host cell defenses.

In order to resolve whether a lack of AnxA2 activity in hBMECs prevented internalization of Sc<Cn*MPR1*>, transmission electron microscopy (TEM) was used to observe hBMECs upon exposure to Sc<Cn*MPR1*>. As expected, TEM micrographs revealed the presence of intercellular tight junctions typical of hBMECs (Figures 6A,B). Nuclei and mitochondria were also observed throughout hBMECs. To assess the role of AnxA2 its activity was blocked by treating hBMECs with a monoclonal antibody of AnxA2. Following a 3 h co-incubation of Sc<Cn*MPR1*> strain and hBMECs, TEM micrographs revealed the presence of Sc<Cn*MPR1*> cells within hBMECs (Figures 6C–F). Sc<Cn*MPR1*> cells appeared as dark oval structures that were in-line with dimensions corresponding to *S. cerevisiae*. Individual Sc<Cn*MPR1*> cells were observed throughout the cytoplasm adjacent to nuclei (Figures 6C–E, labeled as Sc). These results indicated that hBMECs maintained their ability to engulf Sc<Cn*MPR1*> cells despite blocking AnxA2 activity thus strongly suggesting that AnxA2 is not required for association or internalization of Sc<CnMPR1> (Figure 6). Upon closer inspection of the TEM micrographs, we found that the cell surface of Sc<Cn*MPR1*> cells within hBMECs appeared irregular and disrupted likely indicative of cellular damage (Figures 6D,F, indicated by arrows). These striking observations along with the inability of Sc<CnMPR1> cells to cross the hBMECs lacking AnxA2 activity in the *in vitro* model of the BBB, suggested that Sc<CnMPR1> cells were likely trapped within hBMECs since the strain could not transcytose through hBMECs and exit independently of AnxA2 activity. This notion is supported by the transcytosis assays discussed in Figure 5 and by two very recent reports demonstrating that AnxA2 is required for the nonlytic exit of Cn from macrophages and for the movement of Cn across mouse brain endothelial cells (Stukes et al., 2016; Fang et al., 2017).

### AnxA2 and Sc<Cn*MPR1*> co-localize in hBMECs

**Figure 7:**
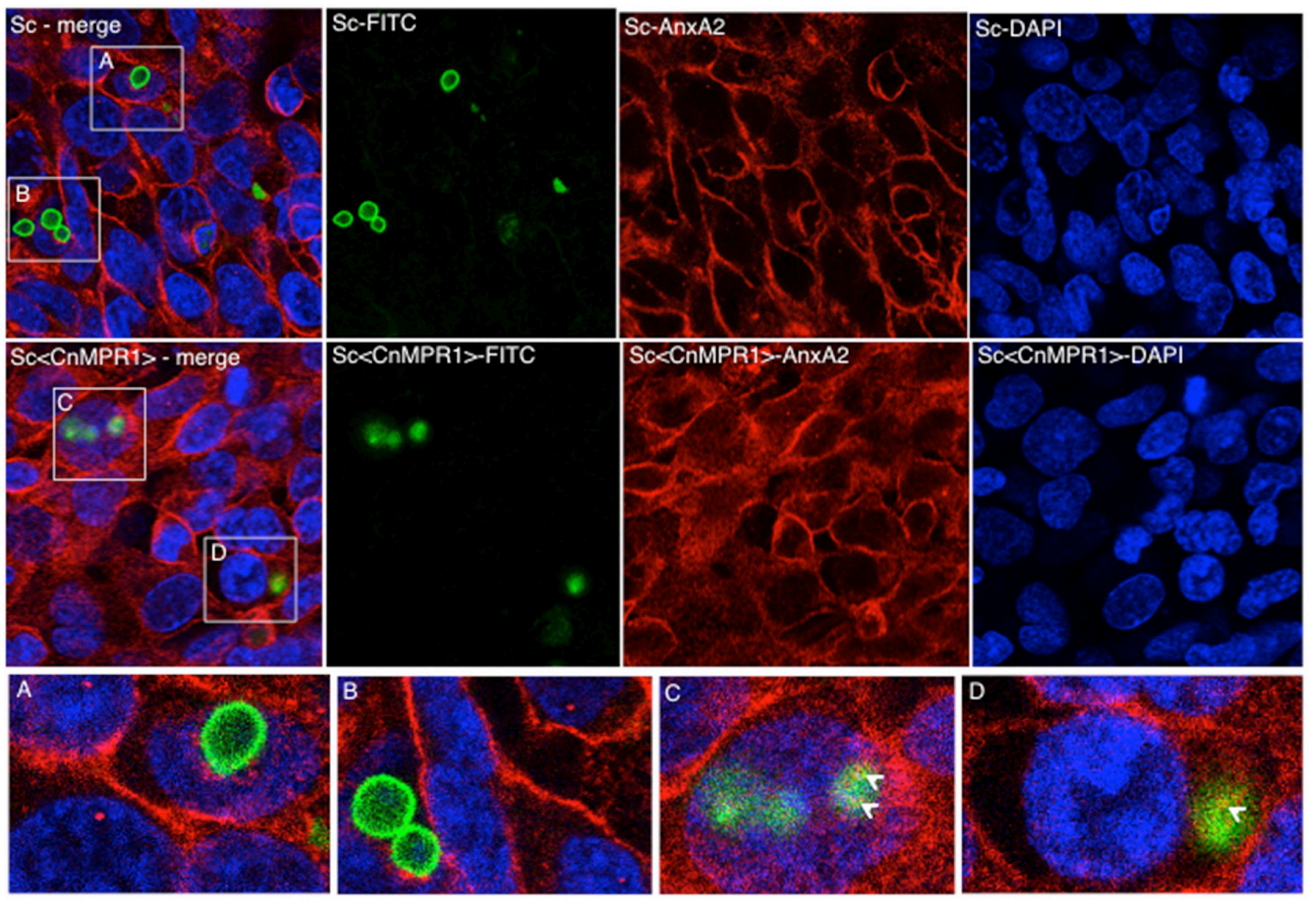
Immunofluorescence (IF) studies demonstrate a re-distribution of AnxA2 from the cell surfaces to the cytosol in hBMECs exposed to Sc<Cn*MPR1*>. HBMECs were challenged with FITC-labeled ScWT (**A,B**; green) or Sc<Cn*MPR1*> (**C,D**; green) for 1.5 h at 37°C with 5% CO_2_. HBMECs were fixed and probed with the anti-AnxA2 primary antibody followed by the Alexa Fluor-555 secondary antibody (red); nuclei were stained with DAPI (blue). **(A,B)** The co-incubation of ScWT and hBMECs revealed that the distribution of AnxA2 was primarily at the cell periphery. Several ScWT cells were observed adjacent to hBMECs but were not internalized by hBMECs and did not co-localize with AnxA2. **(C,D)** hBMECs exposed to Sc<Cn*MPR1*> revealed a re-distribution of AnxA2 to the cytosol and an apparent co-localization with Sc<Cn*MPR1*> (white arrows).

We sought to examine the localization of AnxA2 in hBMECs challenged with either an ScWT strain or the Sc<Cn*MPR1*> strain in order to assess if AnxA2 directly associated with Sc expressing Cn*MPR1* (Figure 7). In hBMECs cells exposed to ScWT, AnxA2 localized primarily to the cell periphery. Several FITC-labeled yeast cells (ScWT) were observed but did not appear to co-localize with AnxA2 nor did they appear to be internalized by hBMECs (Figures 7A,B). In contrast, hBMECs challenged with the Sc expressing Cn*MPR1* (the Sc<CnMPR1> strain) revealed a more diffuse, perinuclear localization pattern for AnxA2, indicative of an increased cytoplasmic presence. FITC-labeled Sc<Cn*MPR1*> cells were observed within hBMECs and appeared to co-localize with AnxA2 (Figures 7C,D, indicated by arrows).

**Figure 8:**
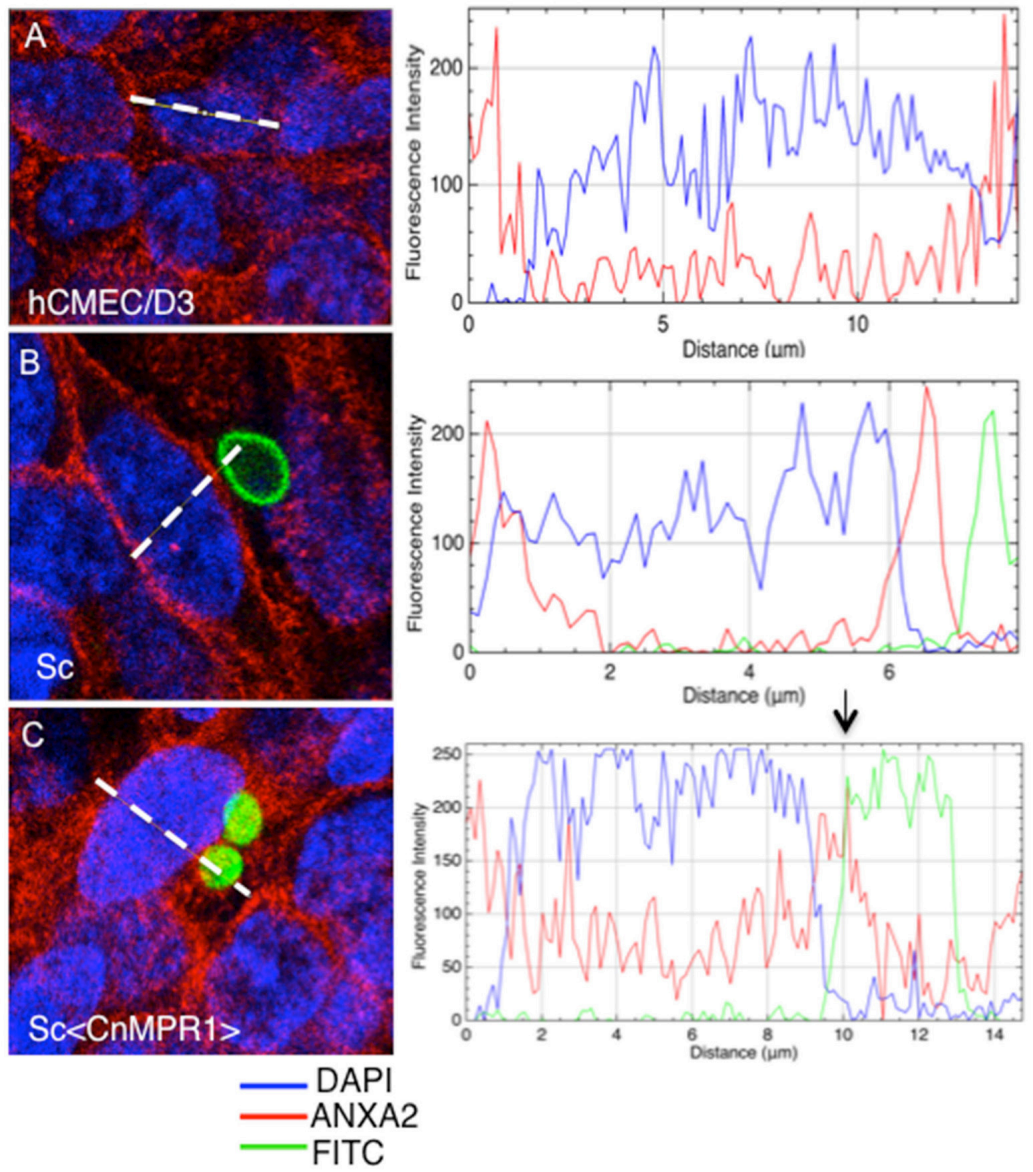
Anx2 co-localizes with Sc<Cn*MPR1*> in the cytosol of hBMECs. HBMECs were challenged, fixed and imaged as described previously. The fluorescence intensity of AnxA2 (red), FITC (yeast cells, green), and DAPI (nuclei, blue) was tracked and graphed (panels on the right-side). **(A,B)** In unchallenged hBMECs or hBMECs challenged with ScWT peak values for fluorescence intensity of AnxA2 revealed a predominantly cell surface localization. **(B)** Fluorescence intensity peaks representing AnxA2, ScWT, or nuclei did not overlap suggesting a lack of co-localization between AnxA2 and ScWT; **(C)** however, a clear overlap between peaks of fluorescence intensity for AnxA2 and FITC-labeled Sc<Cn*MPR1*> cells was detected, suggesting co-localization. Also, fluorescence intensity for AnxA2 was higher along the entire distance of hBMECs challenged with Sc<Cn*MPR1*> suggesting a more diffuse, cytosolic localization for AnxA2.

To further assess an interaction between AnxA2 and Sc<Cn*MPR1*>, the fluorescence intensity was measured across the distance of hBMECs cells (Figure 8). The fluorescence of AnxA2 (red), FITC (yeast cells, green), and DAPI (nuclei, blue) were tracked and graphed. In hBMECs that were either unchallenged (Figure 8A) or challenged with ScWT (Figure 8B), fluorescence intensity graphs revealed a predominantly plasma membrane localization pattern for AnxA2. In addition, the peak values for fluorescence intensity for AnxA2, FITC, and DAPI did not overlap suggesting a lack of co-localization between AnxA2 and ScWT and nuclei (Figure 8B).

In contrast, a clear overlap between peaks of fluorescence intensity for AnxA2 and FITC-labeled Sc<CnMPR1> cells was detected, suggesting co-localization (Figure 8C). In addition, the fluorescence intensity for AnxA2 was higher along the entire distance of hBMECs challenged with Sc<CnMPR1> suggesting a more diffuse, cytosolic localization (Figure 8C); however this was not the case when hBMECs were exposed to ScWT (Figure 8B). Here, the fluorescence intensity peaked at the edges of hBMECs, suggesting a predominantly cell surface localization (Figure 8). Taken together, the data suggest that Sc<Cn*MPR1*> and AnxA2 co-localize in hBMECs and this association is mediated by Mpr1 and is required for a successful transcytosis of Sc<Cn*MPR1*> across hBMECs.

## Discussion

The acquired ability of a strain of *S. cerevisiae* to migrate across hBMECs in an *in vitro* model of the BBB upon the sole expression of Cn*MPR1* was used as a platform for identifying host targets of Mpr1. Initially two working hypotheses were suggested. First, the transmigration of the Sc expressing Cn*MPR1* could be mediated by an altered extracellular architecture of the Sc<Cn*MPR1*> cells via the proteolytic activity of Mpr; however no morphological changes on the surface of Sc<Cn*MPR1*> were observed by SEM analysis. There was no observable phenotypic differences between Sc and Sc<Cn*MPR1*> cells other than the acquired ability to cross the BBB. Also, given that ascomycetes (Sc) and basidiomycetes (Cn) are distantly related, significant differences at the cell surface likely exist and thus it is unlikely that both species would share common targets of Mpr1 (Inglis and Kawchuk, 2002). As previously observed with *C. neoformans*, the strain of Sc expressing Cn*MPR1* migrated across brain endothelial cells independently of tight junction disruption suggesting that both Cn and Sc<Cn*MPR1*> likely crossed the barrier via a similar transcellular mechanism that was dependent on Mpr1 activity (Vu et al., 2014).

Collectively, these observations pointed to a second working hypothesis, that Mpr1 is secreted in order to specifically targets proteins on the surface of hBMECs. The formation of F-actin-mediated ruffles and lamellipodia-like structures on the surface of BMECs exposed to *C. neoformans* has been well described (Chretien et al., 2002; Chen et al., 2003; Chang et al., 2004; Jong et al., 2008; Vu et al., 2009; Huang et al., 2011). These aforementioned surface changes implicate plasma membrane and cytoskeleton remodeling—two processes required during macropinocytosis or phagocytosis. We observed significantly less membrane ruffling in hBMECs when *C. neoformans* lacked Mpr1 and found that cryptococci could not associate with hBMECs in the absence of Mpr1^.^(Vu et al., 2014). The results of the proteomic spectral analysis performed by pulling-down hBMECs surface proteins with Sc<Cn*MPR1*>, revealed that proteins mediating cross-talk between membrane and cytoskeleton reorganization and endocytosis, were specifically targeted by Mpr1. For example, talin, filamin, myosin, profilin, IQGAP1, major vault protein, and AnxA2 were among the proteins with the highest spectral counts. These results support the notion that secreted Mpr1 might promote Sc<Cn*MPR1*) internalization by inducing host cell surface ruffling by targeting cytoskeleton-related proteins. It is known that plasma membrane reorganization and cytoskeleton remodeling are central to the internalization and transcellular movement of fungal cells across the BBB (Chen et al., 2003; Chang et al., 2004; Vu et al., 2013). We previously demonstrated that ANXA2 and S100A10 genes were upregulated in hBMECs following internalization of *C. neoformans*, (Vu et al., 2013) thus we were particularly interested in AnxA2 due to its ability to recruit proteins mediating membrane-actin remodeling and its role in coupling actin dynamics to endocytosis (Gerke et al., 2005).

Typically pathogens enter cells by exploiting endocytic activities of the host in order to move into the cytoplasm and in the case of the BBB, exit on the abluminal side (Gruenberg and van der Goot, 2006). Based on transcytosis assays in the *in vitro* model of the BBB lacking AnxA2 activity and the TEM analysis, we found that AnxA2 was required for the movement of Sc<Cn*MPR1*> across the cytoplasm of the hBMECs; however, AnxA2 did not appear to mediate the association nor the internalization of Sc<Cn*MPR1*> with the BBB. TEM images clearly showed that Sc<Cn*MPR1*> cells had been internalized despite the lack of AnxA2 activity and the host-induced damage to Sc<Cn*MPR1*> cells was likely indicative of an inability to exit hBMECs due to the lack of AnxA2 activity. The extent of the cellular damage observed with Sc<Cn*MPR1*> cells may me unique to Sc since it lacks extracellular features that might be protective in the host environment. For example, in the case of *C. neoformans* the presence of the polysaccharide capsule and melanin in the cell wall have been shown to protect Cn from the onslaught of host cell defenses (Kozel and Gotschlich, 1982; Casadevall et al., 2000; Coelho et al., 2014).

Consistent with our results, a recent study reported a role for AnxA2 in the transcytosis of *C. neoformans* in murine brain endothelial cells (Fang et al., 2017). Our data is also consistent with results demonstrating that the depletion of AnxA2 prevented the biogenesis of multivesicular endosomes from early endosomes and had a direct effect on endocytic transport (Harder and Gerke, 1993; Zobiack et al., 2003; Gerke et al., 2005). Collectively our data suggest that the transcellular movement and exocytosis of Sc<Cn*MPR1*> cells is dependent on membrane trafficking events that involve AnxA2 but these events may be independent from the actions of AnxA2 at the host cell surface. This notion is further supported by recent studies in endothelial cells, where downregulation of AnxA2 blocked calcium-mediated exocytosis of Weibel-Palade bodies and also in macrophages where AnxA2 was also found to promote the nonlytic exocytosis of *C. neoformans* (Konig et al., 1998; Knop et al., 2004; Stukes et al., 2016). We propose that Mpr1 activity likely stimulates the re-organization of the cytoskeleton and in doing so it engages the activity of AnxA2, which then promotes the transcytosis of fungal cells across the BBB.

It is important to consider that the method used here to identify host proteins mediating the fungal-BBB interface has limitations. Some genes are not well expressed in the hCMEC/D3 cell line and thus some protein targets may have been missed (Urich et al., 2012). Although the aim of the study was to focus on surface exposed proteins of hBMECs, we found that a few of the proteins identified may be integral membrane and/or cytosolic proteins. It is conceivable that during the biotin-tagging and purification of endothelial surface proteins, we may have isolated intact protein complexes that included proteins that normally reside below the surface membrane. Nevertheless, compelling data consistent with other reports strongly support a role for AnxA2 in the transcytosis of fungal cells across brain microvascular endothelial cells. Our results further suggest that Mpr1 recruits AnxA2 by likely stimulating membrane and cytoskeleton-reorganization.

## Author contributions

BP and MS performed the LC-MS/MS analysis in the Protoeomics Core Facility. SN performed studies related to material preparation for proteomic analysis, immunofluorescence, and all assays involving the *in vitro* model of the human blood-brain barrier. AG planned and guided the studies, contributed to data analysis, and wrote the manuscript.

### Conflict of interest statement

The authors declare that the research was conducted in the absence of any commercial or financial relationships that could be construed as a potential conflict of interest.
